# Evaluation of Soft Tissue Compensations in Subjects With Facial Asymmetry Using Cone Beam Computed Tomography (CBCT): A Retrospective Study

**DOI:** 10.7759/cureus.52601

**Published:** 2024-01-19

**Authors:** Nisshitha R Setvaji, Srirengalakshmi Muthuswamy Pandian

**Affiliations:** 1 Orthodontics and Dentofacial Orthopaedics, Saveetha Dental College and Hospital, Saveetha Institute of Medical and Technical Sciences (SIMATS) University, Chennai, IND

**Keywords:** compensation, orthognathic surgery, soft tissue, bone tissue, facial asymmetry

## Abstract

Introduction

Facial asymmetry influences aesthetics and can involve either hard or soft tissues or both. Underlying skeletal asymmetry can be compensated by differential expression of soft tissue thickness on either side. Orthognathic surgical planning needs to take the interaction between the hard and soft tissues into account. The aim of this study was to assess the bilateral thickness of hard tissues and the corresponding facial soft tissue in asymmetric subjects to assess the compensation using cone beam computed tomography (CBCT) imaging.

Materials and methods

CBCT measurements of 30 skeletal Class l asymmetric untreated patients with menton deviation greater than 4 mm were included in the study. The side towards which the menton deviated was considered as the deviated side and taken as the control group (GC). The contralateral side of the menton deviation was considered as the non-deviated side and was taken as the test group (GT). The greatest width of both hard and soft tissues was measured at the head of the condyle; the centre of the ramal upper, middle, and lower thirds; furcation of the first molar; and apices of the first premolar and canine. Each landmark was precisely positioned on all three planes and the measurements were correlated. An independent t-test compared the difference of both hard and soft tissues between deviated and non-deviated sides. The correlation between the hard and soft tissues of both non-deviated and deviated sides was performed using the Pearson correlation two-tailed test.

Results

In the condylar and mid ramal regions, significant differences between the hard and soft tissues were noted in the GT (p < 0.05). In the non-deviated side, at the condylar region, it was noted that with an increase in hard tissue thickness, there was a decrease in soft tissue thickness, while in mid and lower ramal regions, it was noted that with a decrease in hard tissue thickness, there was an increase in soft tissue thickness. No significant difference was seen in the tooth-bearing section of the mandible (p > 0.05). Pearson’s correlation showed a highly significant negative correlation between the hard and soft tissues of the GT at the level of the condyle and the ramus (p < 0.05). Non-significant correlation was seen between the hard and soft tissues at the molar, premolar and canine areas of the GT. No significant correlation between the hard and soft tissues was seen at any level in the GC (p > 0.05).

Conclusion

In the non-deviated side, the non-tooth-bearing segment of the mandible (condyle and ramus) showed differences between the hard and soft tissue thicknesses. With an increase in the hard tissue thickness, there was a corresponding decrease in the soft tissue thickness and vice versa which is attributed as compensation. The tooth-bearing segment of the non-deviated side did not show compensation. There is no compensation seen on the deviated side in both segments.

## Introduction

Facial aesthetics have a direct influence on an individual’s quality of life. Harmony between the numerous components of the craniofacial complex is necessary to achieve facial aesthetics. Facial asymmetry negatively impacts functional and facial aesthetics. Correcting facial asymmetry is a key objective of orthognathic surgery and orthodontic treatment. Most subjects in a given population have been reported to have some degree of craniofacial asymmetries, even those discerned to be normal. Correcting any apparent asymmetry may enhance social interactions and interpersonal relationships. 

Previous studies have shown a significant preference for symmetric faces, considering them to be more attractive [1,2]. Various theories have been proposed over time attempting to make beauty or attractiveness measurable. Seghers et al. first mentioned the use of the golden proportion in aesthetic facial surgery [3]. Ricketts was the first orthodontist who used the divine proportion or Fibonacci sequence for scientific rather than subjective perceptions of aesthetics. Previous facial metric studies by Grammer and Thornhill [2] and Jones et al. [4] and psychophysical measures by Mealey et al. [5] and Penton-Voak et al. [6] provide evidence that naturally occurring symmetrical faces are attractive and receive positive judgements. Two concepts have been proposed to describe the universal human inclination for symmetry. According to the Evolutionary Advantage theory, symmetrical faces are viewed as more attractive because they signify an individual’s good health. The perceptual bias theory states that all symmetrical stimuli are more readily processed by the visual system [1].

Patients with facial asymmetry are evaluated through clinical assessment, photography, cephalography, ultrasonography, laser scanners, computed tomography (CT) and cone beam computed tomography (CBCT). Cephalometric measurements ascertain skeletal symmetry; however, subjective evaluations such as the discernment of facial asymmetry are determined by soft tissue features and facial outlines. Results from soft tissue analyses based on cephalometric evaluations of facial asymmetries may differ from skeletal analyses of the same. Asymmetry in the face can be affected by either hard or soft tissue, or both. Underlying skeletal asymmetry may be compensated by differential expression of soft tissue thickness on either side of the face [7]. Due to the multifactorial aetiology of facial asymmetry including asymmetric muscular habits such as unilateral mastication, facial muscles may develop differently on either side, highlighting or hiding bone asymmetry [8]. The clinician occasionally neglects to evaluate facial asymmetry, despite it being a crucial component of a correct orthodontic diagnosis.

The lower third of the face is where facial asymmetry is most frequently found. Severt and Proffit showed that only 5% of the observed subjects had facial asymmetry in their upper face, 36% in their midface and 74% in their lower face [9]. If the deviation of the menton is greater than 4 mm, it can be recognised as facial asymmetry [10]. Haraguchi et al. and Chebib and Chamma have reported left-sided menton deviation as more common than right-sided deviation [11,12]. An asymmetry of 3 mm was regarded as abnormal for smiling. Asymmetry is considered to exist when there is a difference of more than 3 mm between the eyebrow and oral commissure. Lee et al. proposed applying these as diagnostic criteria for facial asymmetry [7].

Mccance et al. [13] contended that facial asymmetries are evident upon clinical observations. Shah and Joshi [14] reported that overlying soft tissues compensate for underlying hard tissue asymmetry. Furthermore, when analysed on posteroanterior radiographs, patients who are clinically classified as symmetric or mildly asymmetric may actually have profound skeletal asymmetries, according to Masuoka et al. [10].

Various two-dimensional (2D) diagnostic tools present with magnification, distortion and superimposition problems. The evolution of three-dimensional (3D) tools such as CBCT allows for better understanding of facial structures, overcoming the limitations of 2D diagnostic tools. It is pertinent for orthodontists to pay close attention to the interaction between the hard and soft tissues when performing orthognathic surgery on asymmetrical patients, so that the treatment recommendations take into account facial appearance as well as skeletal structures [15]. Therefore, the aim of this study was to assess the bilateral thickness of hard tissues and the corresponding facial soft tissue in asymmetric subjects to assess the compensation using CBCT imaging.

## Materials and methods

Study setting

This was a retrospective CBCT study conducted at Saveetha Dental College and Hospital, Chennai, Tamil Nadu, between March 2022 and April 2022. An institutional review board ethical clearance (IHEC/SDC/ORTHO-2105/22/199) was obtained prior to starting this study.

Study sample

Based on a previous study published by Huang et al., the sample size for this study was estimated to be 25 [16]. The inclusion criteria consisted of CBCTs of patients diagnosed clinically with skeletal asymmetry based on the deviation of the menton from the facial midline. If the deviation at centric occlusion was found to be equal to or greater than 4 mm, the patient was considered to have asymmetry and was included in the study [10]. The study included CBCTs of patients with a Class I skeletal pattern with a fully erupted permanent dentition and an average growth pattern with no history of orthodontic or surgical treatment. Patients with syndromes, craniofacial defects, trauma, infection, degenerative diseases of the TMJ and functional deviation at occlusion were excluded from the study. Patients of horizontal or vertical growth patterns were also excluded as the difference in their muscular pattern would introduce bias [17,18].

**Figure 1:**
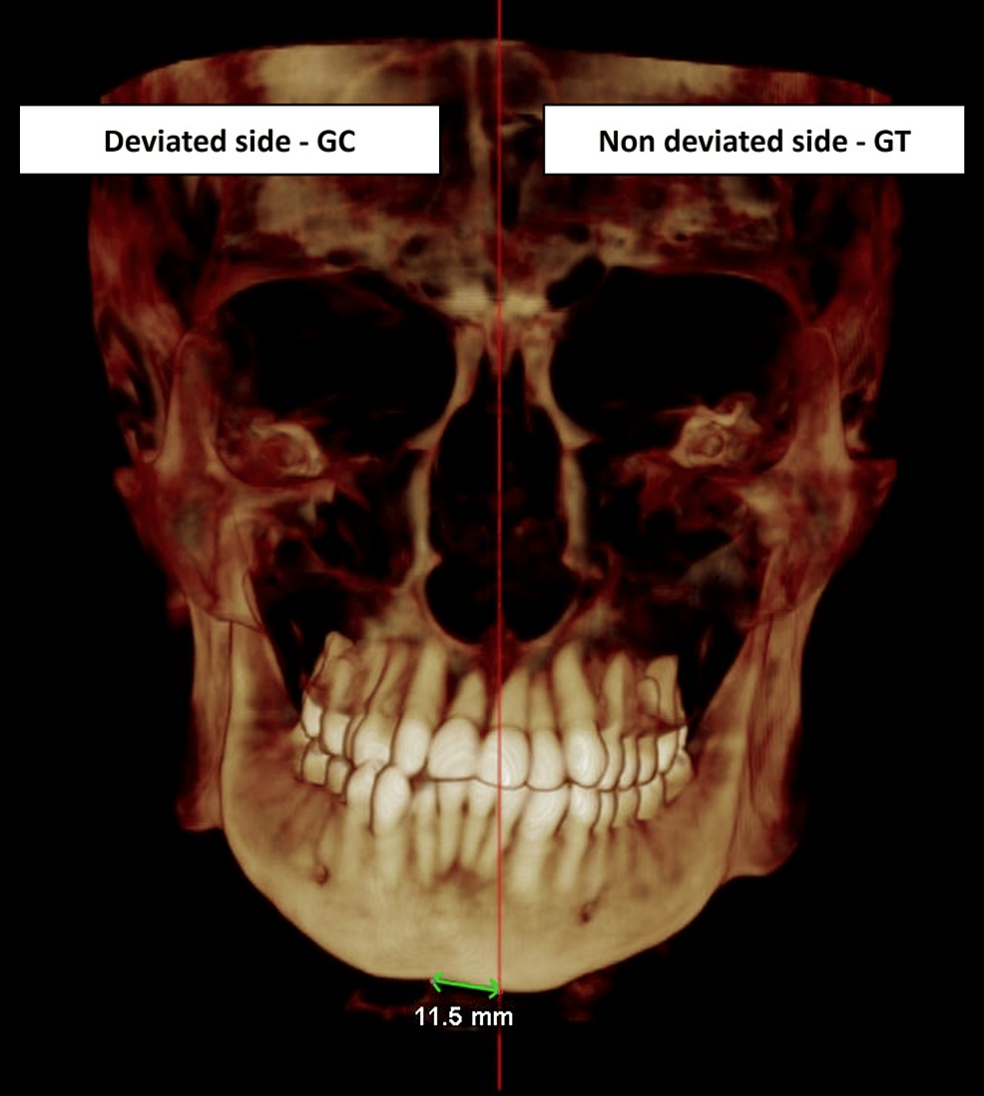
Image depicting the control group (GC) and test group (GT)

Based on the inclusion and exclusion criteria, CBCT records of 30 patients were selected for analysis. The side towards which the menton deviated was considered the deviated side and taken as the control group (GC). The contralateral side of the menton deviation was considered the non-deviated side and was taken as the test group (GT) (Figure 1). 

CBCT image and parameters measured

The CBCT of the skull for each subject was taken using the KODAK 9500 Cone Beam 3D System (Carestream Health, Rochester, USA) following the standard regulation of 120 kVp, 4 mA and 0.3 mm voxel size, field of view of 8/5 mm and exposure time of 24 seconds [15]. All the CBCT scans were standardised and recorded with each subject standing in their natural head position, teeth in centric occlusion with lips in a relaxed position. The CBCT scans were exported in DICOM format and evaluated using Dolphin Imaging software (version 11.7). On all CBCT scans, the anatomical structures were determined and measured bilaterally by the same operator following a standard protocol and reverified by the supervisor at a one-week interval. The anatomic landmarks taken into consideration were the greatest width of head of the condyle; widths at the centre of the ramal upper, middle and lower thirds; width at the furcation of the first molar; width at apex of the first premolar; and width at the apex of the canine. The hard tissue thickness was measured along the greatest width of the particular structure and the corresponding soft tissue thickness was measured (Table 1).

**Table 1:** Hard and soft tissue parameters included in the study CBCT: Cone beam computed tomography

Parameter	Measurement
Condyle (hard tissue)	Maximum width of the condylar head as seen through CBCT sections
Condyle (soft tissue)	Corresponding soft tissue measurement of maximum width of condylar head
Ramus (hard tissue)	The total length of the ramus was measured from the deepest point on the sigmoid notch to the antegonial point. The total distance was divided by 3 to be considered as 1/3, 2/3 and 3/3 ramus
Ramus (soft tissue)	Corresponding soft tissue measurement of the maximum width of the ramus
Molar (hard tissue)	Hard tissue measurement taken at the furcation level of the mandibular first molar
Molar (soft tissue)	Corresponding soft tissue measurement of the maximum width at the furcation level of the mandibular first molar
Premolar (hard tissue)	Hard tissue measurement taken at the apex level of the mandibular first premolar
Premolar (soft tissue)	Corresponding soft tissue measurement of the maximum width at the apex of the mandibular first premolar
Canine (hard tissue)	Hard tissue measurement taken at the apex level of the mandibular canine
Canine (soft tissue)	Corresponding soft tissue measurement of the maximum width at the apex of the mandibular canine

**Figure 2:**
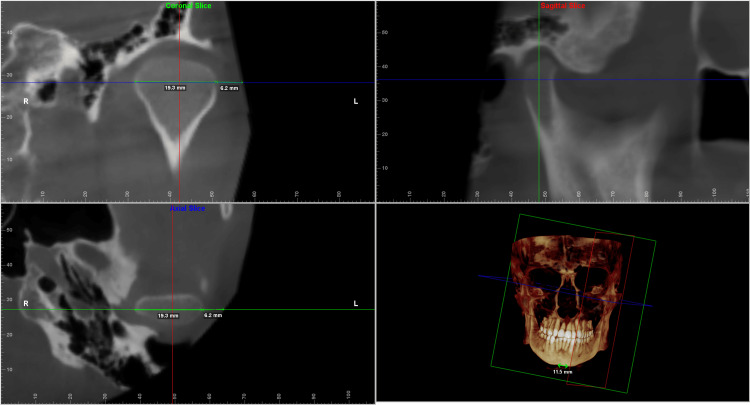
Multiplanar reconstructed images produced using Dolphin Imaging software depicting the measurements taken at the condylar level in all the three slices (coronal, axial and sagittal) along with the reconstructed view

**Figure 3:**
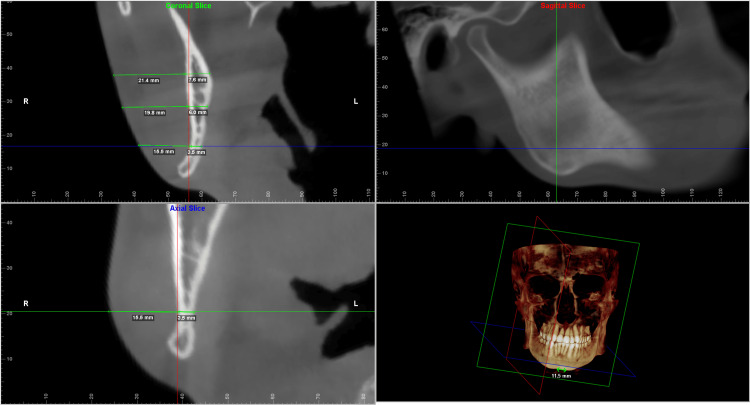
Multiplanar reconstructed images produced using Dolphin Imaging software depicting the measurements taken at the three levels in the ramal region. It shows the measurements in all the three slices (coronal, axial and sagittal) along with the reconstructed view

**Figure 4:**
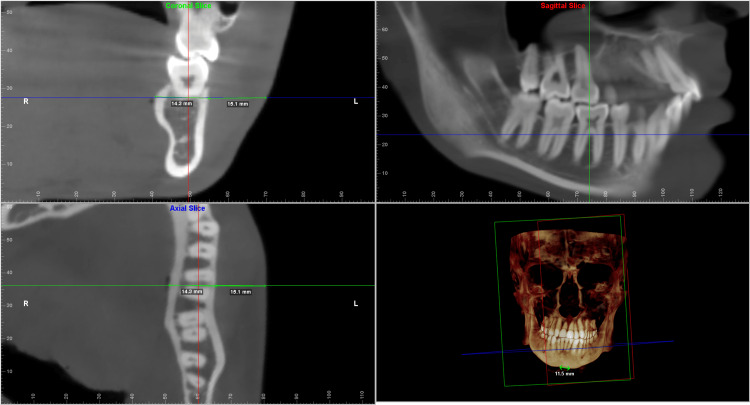
Multiplanar reconstructed images produced using Dolphin Imaging software depicting the measurements taken at the molar furcation level in all the three slices (coronal, axial and sagittal) along with the reconstructed view

**Figure 5:**
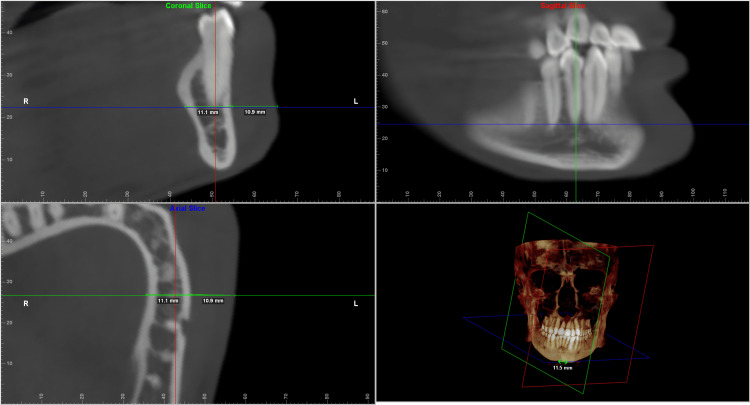
Multiplanar reconstructed images produced using Dolphin Imaging software depicting the measurements taken at the premolar apex in all the three slices (coronal, axial and sagittal) along with the reconstructed view

**Figure 6:**
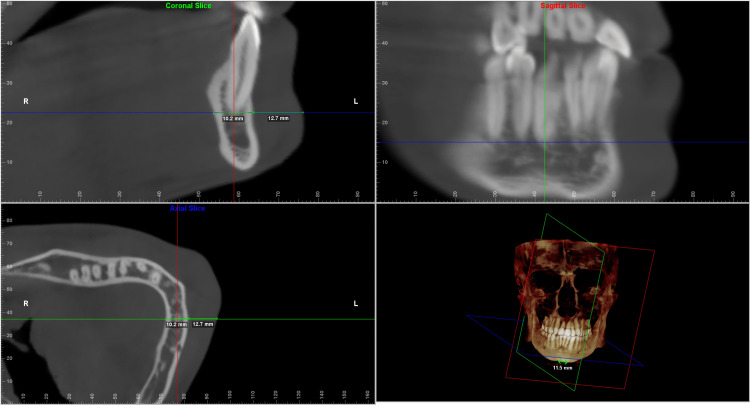
Multiplanar reconstructed images produced using Dolphin Imaging software depicting the measurements taken at the canine apex in all the three slices (coronal, axial and sagittal) along with the reconstructed view

Each of the landmarks was precisely positioned on all three reference planes and was found to correlate on all three slices. The values were taken for both the deviated and the non-deviated side of the face using the digitise/measure tool of the Dolphin Imaging software. The greatest width was then measured along the standard reference plane to determine the hard tissue and soft tissue thickness. The values measured were verified to be identical on both the coronal and the axial sections (Figures 2-6). 

The rationale behind the landmarks chosen for the study were areas corresponding to the insertion of muscles of mastication. The width of the condyle corresponds to the insertion of lateral pterygoid. The widths at the centre of the ramal upper, middle and lower thirds correspond to the insertion of the masseter and medial pterygoid on either side of the ramus. Furcation of the first molar, apex of the first premolar and apex of the canine represent those areas away from the masticatory muscles. The landmarks were also grouped as tooth-bearing areas and non-tooth-bearing areas to investigate the role of dentition in the development of asymmetry. All the CBCT measurements were repeated after an interval of three weeks for evaluation of errors in landmark identification.

Statistical analysis

IBM SPSS Statistics for Windows, Version 23.0 (Released 2011; IBM Corp., Armonk, New York, United States) was used for performing the statistical analyses. Descriptive analysis was done for all the measured parameters. Data normality was determined by the Shapiro-Wilk test which presented a normal distribution. An independent t-test compared the difference of both hard and soft tissues between deviated and non-deviated sides (p < 0.05). Correlation between the hard and soft tissues of both deviated and non-deviated sides was performed using the Pearson Correlation two-tailed test. To evaluate the intraobserver reliability and method errors, the Wilcoxon test and intraclass correlation coefficient were used. The statistical significance level was set at p < 0.05.

## Results

The Shapiro-Wilk test found a normal distribution of data. An independent sample t-test was carried out to test the intergroup differences between the hard and soft tissues between GT and GC. Significant intergroup differences were noted at the condyle (p = 0.049, 0.020) and the upper (p = 0.032, 0.019), middle (p = 0.033, 0.048) and lower (p = 0.007, 0.044) thirds of the ramus for all hard and soft tissues. This shows a difference in the thickness of the structures leading to asymmetry at the condylar and ramal regions. No significant difference was seen at the level of the first molar furcation (p = 0.121, 0.718), the first premolar apex (p = 0.896, 0.623) and the canine apex (p = 0.761, 0.851). The results of the independent sample t-tests are tabulated in Table 2.

**Table 2:** Independent sample t-test The data has been represented as Mean ± SD in mm. p < 0.05

Landmark	Non-deviated Side (Mean + SD) (mm)	Deviated Side (Mean + SD) (mm)	p-value
Hard Tissue Thickness			
Condyle – hard tissue	18.38 ± 2.35	16.98 ± 2.12	0.049
1/3 Ramus – hard tissue	5.41 ± 1.69	5.96 ± 1.88	0.032
2/3 Ramus – hard tissue	6.79 ± 2.17	7.40 ± 2.13	0.033
3/3 Ramus – hard tissue	5.28 ± 1.54	5.45 ± 1.23	0.007
Molar – hard tissue	11.18 ± 1.15	10.44 ± 1.74	0.121
Premolar – hard tissue	8.60 ± 1.35	8.53 ± 1.95	0.896
Canine – hard tissue	7.81 ± 1.69	7.65 ± 1.61	0.761
Soft Tissue Thickness			
Condyle – soft tissue	9.13 ± 1.76	10.21 ± 3.54	0.020
1/3 Ramus – soft tissue	17.39 ± 3.27	18.30 ± 2.98	0.019
2/3 Ramus – soft tissue	17.43 ± 2.98	16.57 ± 3.35	0.048
3/3 Ramus – soft tissue	14.00 ± 3.80	13.43 ± 3.17	0.044
Premolar – soft tissue	11.73 ± 3.27	11.29 ± 2.26	0.623
Molar – soft tissue	14.50 ± 5.22	14.99 ± 2.96	0.718
Canine – soft tissue	11.52 ± 1.44	11.43 ± 1.57	0.851

Pearson’s correlation test was carried out to test the correlation between the hard and soft tissues of the GT and GC. The test revealed a highly significant negative correlation between both hard and soft tissues in the GT at the level of the condyle (p = 0.048, R = -0.477) and upper (p = 0.016, R = -0.0529), middle (p = 0.000, R = -0.899) and lower (p = 0.000, R = -0.729) thirds of the ramus. In the non-deviated side, at the condylar region, it was noted that with an increase in the hard tissue thickness, there was a decrease in the soft tissue thickness. In the mid and lower ramal regions, it was noted that with a decrease in the hard tissue thickness, there was an increase in the soft tissue thickness. No significant correlation was seen at the level of the first molar furcation (p = 0.545, R = 0.144), the first premolar apex (p = 0.722, 0.085) and the canine apex (p = 0.151, R = -0.333) in the GT. No significant correlation between the hard and soft tissues was seen at any level in the GC (p > 0.05). Descriptive statistics and Pearson’s correlation values along with their significance are tabulated in Table 3.

**Table 3:** Results of descriptive statistics and Pearson’s correlation with its significance GT: Test group; GC: control group The data has been represented as Mean ± SD in mm and Pearson's correlation coefficient (R). p < 0.05

Landmark	Mean ± SD (mm)	Pearson’s Correlation Coefficient (R)	p-value
Non-deviated Side (GT)			
Condyle – hard tissue	18.38 ± 2.35	-0.477	0.048
Condyle – soft tissue	9.13 ± 1.76		
1/3 Ramus – hard Tissue	5.41 ± 1.69	-0.529	0.016
1/3 Ramus – soft Tissue	17.39 ± 3.27		
2/3 Ramus – hard tissue	6.79 ± 2.17	-0.899	0.000
2/3 Ramus – soft tissue	17.43 ±2.98		
3/3 Ramus – hard tissue	5.28 ± 1.54	-0.729	0.000
3/3 Ramus – soft tissue	14.00 ± 3.80		
Molar – hard tissue	11.18 ± 1.15	0.144	0.545
Molar – soft tissue	14.50 ± 5.22		
Premolar – hard tissue	8.60 ± 1.35	0.085	0.722
Premolar – soft tissue	11.73 ± 3.27		
Canine – hard tissue	7.81 ± 1.69	-0.333	0.151
Canine – soft tissue	11.52 ± 1.44		
Deviated Side (GC)			
Condyle – hard tissue	16.98 ± 2.12	-0.251	0.287
Condyle – soft tissue	10.21 ± 3.54		
1/3 Ramus – hard tissue	5.96 ± 1.88	0.001	0.997
1/3 Ramus – soft tissue	18.30 ± 2.98		
2/3 Ramus – hard tissue	7.40 ± 2.13	-0.551	0.012
2/3 Ramus – soft tissue	16.57 ± 3.35		
3/3 Ramus – hard tissue	5.45 ± 1.23	-0.182	0.443
3/3 Ramus – soft tissue	13.43 ± 3.17		
Molar – hard tissue	10.44 ± 1.74	-0.130	0.585
Molar – soft tissue	14.99 ± 2.96		
Premolar – hard tissue	8.53 ± 1.95	0.393	0.086
Premolar – soft tissue	11.29 ± 2.26		
Canine – hard tissue	7.65 ± 1.61	-0.195	0.409
Canine – soft tissue	11.43 ± 1.57		

## Discussion

Facial asymmetry when associated with severe malformations requires a multidisciplinary approach. Several factors affect the effectiveness of treatment and impede the achievement of absolute symmetry. It is crucial to understand the behaviour of soft tissue with respect to changes in the underlying hard tissue. The landmarks chosen for the study include areas corresponding to the insertion of the muscles of mastication which were grouped into non-dentition segment areas and the remaining as dentition segment areas. Kim et al., Ariji et al., Weijs WA and van Spronsen et al. reported that the muscles of mastication play a determining role in facial asymmetry [19-23]. The ramal area is crucial for investigation as it forms the bilateral contour defining the face which impacts the perception of facial symmetry [24].

Non-dental segment

Condyle

In the present study, a negative correlation established between the hard and soft tissues at the greatest width of the condyle suggests that the soft tissues do compensate for the underlying skeletal asymmetry. In a study by Chou et al. [25], differences in the axial condylar angle and condylar volume were observed in Class III patients with facial asymmetry which is in accordance with the present results, where we report a difference in the thickness of the condyles bilaterally compensated by the overlying soft tissue at the condylar region.

Ramus

Previous studies by Yeung et al. and Tam et al. reported differences in the ramal thickness in asymmetric patients [24,26]. The upper third thickness can be influenced by temporalis muscle insertion into the coronoid process, whereas the middle and lower third can be influenced by the masseter and medial pterygoid muscle activity. Our study also reported that the ramal widths measured at the upper, middle and lower thirds showed a significant difference between the hard and soft tissue thickness. The soft tissue thickness decreased with an increase in the hard tissue thickness. This important finding could have implications while planning sagittal split osteotomy or orthomorphic surgery for the correction of facial asymmetry.

Dental segment

Molar Furcation

The molar represents the region with the highest occlusal loading with other perioral musculature attachments. A study by Hikosaka et al. reported volumetric changes in the body of the mandible of asymmetric patients [27]. However, in this study, no difference in the thickness was reported between the hard tissue and the soft tissue of the deviated and non-deviated sides. There was also no significant correlation between the hard and soft tissues on both sides at the furcation of the first molar. No soft tissue compensation was appreciated.

Premolar and Canine Apex

The apices of the first premolar and canine lie closely related to the suprahyoid and genial muscles. In comparison with the hard and soft tissue thickness of this region, no correlation was seen. The soft tissue did not compensate for the underlying skeletal disorder along the body of the mandible.

It is necessary to maintain the mechanical performance of the masticatory apparatus, which in turn is compensated by the facial balance maintained between the facial hard and soft tissue thicknesses at the condylar and ramal (non-dental) regions. No correlation was seen between the hard and soft tissues of the dental segment of the mandible indicating a dentoalveolar compensation seen irrespective of the facial asymmetry which may have multiple aetiologies. The alveolar structures which act as microskeletal units are responsible for the development of the dentition and its associated substructures [28]. However, the alveolus does not bear the significant effects of the capsular matrix (which in this case is the facial soft tissues). Hence, this could explain the possible lack of correlation between the soft and hard tissue thicknesses at the dental segment of the mandible. 

This study differs from the previous studies in the sections taken for measurement. Each landmark was precisely positioned along the three reference planes, and the greatest width was measured. The values were found to correlate on both coronal and axial sections to give the true width of the hard and soft tissues. The previous similar studies measured the distance from the centre of the midline without accounting for the true width of the structure, probably leading to bias in their measurements [15,24].

The results of the present study also correlate with the results from the previous studies by Lee et al. [29], Kim et al. [30] and Huang et al. [16] who observed thicker hard tissue on the deviated side which was compensated by thinner soft tissue. Thus, soft tissue camouflages the underlying hard tissue asymmetry. However, studies by Tam et al. [24] and Siqueira de Lima et al. [15] contradict the findings of this study. They report insufficient evidence as to whether soft tissue can truly camouflage underlying skeletal asymmetry. This may be owed to their methodological variations and inclusion of only borderline asymmetrical patients. More randomised controlled trials are required to validate the present findings.

A limitation of this study is that factors such as ageing and body mass of the soft tissues were not taken into consideration. Another limitation is that only the asymmetry with menton deviation was considered, whereas the asymmetry of other hard tissue landmarks may exhibit different soft tissue compensation. Other than the underlying skeletal asymmetry, various muscular factors may affect the soft tissue behaviour, and hence, further research is required to better understand this subject matter.

## Conclusions

In individuals with asymmetry, the non-tooth-bearing segment of the mandible (condyle and ramus) shows soft tissue compensation on the non-deviated side. The tooth-bearing segment of the mandible does not get compensated by the soft tissue on the non-deviated side. There is no compensation seen on the deviated side in both segments. These findings should be applied in the treatment planning principles when diagnosing and treating a case with asymmetry to help us comprehend the behaviour of soft tissue after treatment and may provide greater predictability of treatment outcomes.
